# The complete mitochondrial genome of the firefly, *Luciola curtithorax* (Coleoptera: Lampyridae)

**DOI:** 10.1080/23802359.2018.1437817

**Published:** 2018-03-15

**Authors:** Jinfeng Hu, Xinhua Fu

**Affiliations:** a Fujian Key laboratory for Monitoring and Integrated Management of Crop Pest, Institute of Plant Protection, Fujian Academy of Agricultural Science, Fuzhou, Fujian, China;; b Hubei Insect Resources Utilization and Sustainable Pest Management Key Laboratory, College of Plant Science and Technology, Huazhong Agricultural University, Wuhan, Hubei, China;; c Firefly Conservation Research Centre, Wuhan, Hubei, China

**Keywords:** *Luciola curtithorax*, firefly, Lampyridae, mitochondrial genome

## Abstract

We report the complete mitochondrial genome of firefly, *Luciola curtithorax*. The circular genome of 16,882 bp has a base composition of A (44.98%), C (11.84%), G (8.15%), and T (35.03%). Our sequence is similar to other Metazoa, which contains 13 protein-coding genes. All 13 protein-coding genes were initiated by the ATN (ATT, ATA, and ATG) codon. Ten protein-coding genes stopped with TAA or TAG codon and the other three genes have an incomplete termination codon, a single T––. We sequenced the mitochondrial genome of fireflies to analyze phylogenetic relationships and deduce the evolution of their flashing signals.

Fireflies are a family of insects in the beetle order Coleoptera. Using morphological characters, 12 species of genus *Luciola* had known from Taiwan (Matsumura 1928; Pic 1928; Jeng et al. 2003). *Luciola curtithorax* Pic, 1928 is found in Taiwan, China (Pic 1928). It is distributed from Vietnam, and it is also widely distributed in adjacent Chinese provinces of Hainan, Hubei, Hongkong, and Taiwan (Fu 2014).

Mitochondrial genome sequences are essential to a comprehensive understanding of the evolution of Lampyridae and other luminescent beetles (Ermakov et al. 2006). Here, we elucidate the mtDNA genome of *L. curtithorax*.

These male fireflies used in this study were collected from Xiapu Town, Tongshan County, Hubei Province, China, in 13 July 2016, and were stored in Natural History Museum, Huazhong Agricultural University, Wuhan, Hubei, China (its accession no. is LC2016071301). *L. curtithorax*’s habits, flashing signals, and some morphology have been studied in detail (Pic 1928). However, there is no genetic research information about *L. curtithorax*.

Specific primers were designed based on these conserved regions sequences. The PCR reaction was carried out with LA Taq polymerase for 35 cycles at 94 °C for 30 s, and annealed at 50 °C for 30 s, followed by extension at 72 °C for 1 min per 1 kb. Sequences were assembled using the software DNAstar v7.1 (Madison, WI) and adjusted manually to generate the complete sequence of mitochondrial DNA.

The complete mitochondrial genome sequence of *L. curtithorax* (GenBank MG770613) has 16,882 bp and has a base composition of A (44.98%), C (11.84%), G (8.15%), and T (35.03%). Similar to other Metazoa, our sequence contains 13 protein-coding genes, 22 transfer RNA genes, two ribosomal RNA genes, and a non-coding AT-rich region, which represents a typical insect mitochondrial genome (Wolstenholme 1992). The open frames of the 13 protein-coding genes were inferred from three other fireflies:*Aquatica leii*, *L. substriata*, and *Pyrocoelia rufa* (Lee et al. 2004; Jiao et al. 2013; Mu et al. 2015). All 13 PGGs initiated with ATN (ATT, ATA, and ATG) codon. Among those genes, 6 PCGs (*COII*, *ATP6*, *COIII*, *ND4*, *ND4L*, and *CYTB*) initiate from ATG, and four PCGs (*COI*, *ATP8*, *ND6*, and *ND1*) initiate from ATT, and three PCGs (*ND2*, *ND3*, and *ND5*) start with ATA. Seven PCGs are terminated with TAA (*ND2*, *COI*, *ATP8*, *ATP6*, *ND4*, *ND4L*, and *ND6*). Three ended with TAG (*ND3*, *CYTB*, and *ND1*). And three PCGs (*COII*, *COIII*, and *ND5*) terminate with incomplete stop codon T––. The 12S and 16S ribosomal RNA genes are 743 and 1263 bp, respectively. The length of the 22 tRNA genes ranged from 62 to 84 bp. The AT-rich region is 2267 bp.

The phylogenetic tree among the eight species based on mitochondrial genome sequences were aligned in MEGA 5 (Phoenix, AZ) (with 1000 bootstrap replicates) to construct a Neighbour-Joining tree (Figure 1).

**Figure 1.**
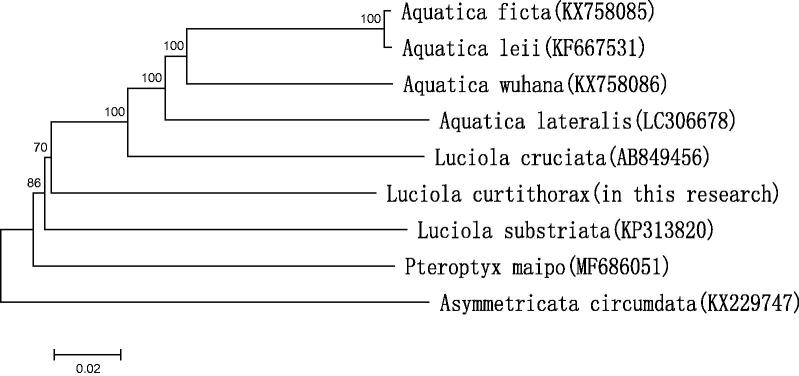
Molecular phylogeny of *L. curtithorax* and eight other firefly species based on the complete mitochondrial genome. The complete mitochondrial genome was downloaded from GenBank and the phylogenetic tree was constructed by neighbour-joining method with 1000 bootstrap replicates. MtDNA accession numbers used for tree construction are as follows: *A. ficta* (KX758085), *A. leii* (KF667531), *A. wuhana* (KX758086), *L. cruciata* (AB849456), *Asymmetricata circumdata* (KX229747), *A. lateralis* (LC306678), *Pteroptyx maipo* (MF686051), and *L. substriata* (recently identified as *Sclerotia flavida* by Ballantyne et al. (2016) (KP313820).

The result shows that *L. curtithorax* is most closely related to *L. cruciata*, which belongs to an entirely different genus in the Lampyridae.

In conclusion, the complete mitochondrial genome sequence of *L. curtithorax* provides an important molecular framework for further phylogenetic analyses of fireflies. These data are essential for comprehensive understanding of the role of sexual and natural selection in the evolution of firefly flashing signals.
